# Homologous Recombination under the Single-Molecule Fluorescence Microscope

**DOI:** 10.3390/ijms20236102

**Published:** 2019-12-03

**Authors:** Dalton R. Gibbs, Soma Dhakal

**Affiliations:** Department of Chemistry, Virginia Commonwealth University, 1001 West Main Street, Richmond, VA 23284, USA; gibbsdr@vcu.edu

**Keywords:** single-molecule fluorescence microscopy, fluorescence resonance energy transfer (FRET), homologous recombination (HR), DNA break repair, DNA curtain, optical tweezers, Holliday junction (HJ)

## Abstract

Homologous recombination (HR) is a complex biological process and is central to meiosis and for repair of DNA double-strand breaks. Although the HR process has been the subject of intensive study for more than three decades, the complex protein–protein and protein–DNA interactions during HR present a significant challenge for determining the molecular mechanism(s) of the process. This knowledge gap is largely because of the dynamic interactions between HR proteins and DNA which is difficult to capture by routine biochemical or structural biology methods. In recent years, single-molecule fluorescence microscopy has been a popular method in the field of HR to visualize these complex and dynamic interactions at high spatiotemporal resolution, revealing mechanistic insights of the process. In this review, we describe recent efforts that employ single-molecule fluorescence microscopy to investigate protein–protein and protein–DNA interactions operating on three key DNA-substrates: single-stranded DNA (ssDNA), double-stranded DNA (dsDNA), and four-way DNA called Holliday junction (HJ). We also outline the technological advances and several key insights revealed by these studies in terms of protein assembly on these DNA substrates and highlight the foreseeable promise of single-molecule fluorescence microscopy in advancing our understanding of homologous recombination.

## 1. Introduction

Homologous recombination (HR) is a well-conserved and essential biological process for both genetic exchange and the repair of double-stranded DNA (dsDNA) breaks [1,2,3,4]. The HR process has been a hot research topic for decades, initially due to the quest for unraveling the fundamental mechanisms of HR, its role in DNA replication, and in rescuing stalled replication forks [1,5]. Later HR’s role in DNA damage repair rose to significance, due to the fact that double-stranded DNA breaks are a form of DNA lesion that, if left unrepaired, can lead to genetic instability, genetic defects, and certain cancers which, as a consequence, is highly dangerous to cells [1,6,7]. The details of the HR process including DNA mismatch repair have been previously discussed [1,8,9] and reviews covering the entire single-molecule perspective on HR, including studies using force microcopy and tethered particle motion, have been published [7,10,11]. However, the focus of these previous reviews/surveys has been from a biological perspective, detailing knowledge of the HR pathway. The single-molecule fluorescence microscopy studies of HR have been quite extensive, but these studies thus far are limited to only a few labs across the world. Therefore, in this review, we intended to bring this area of study to focus as an effort to allow current and new researchers to realize the capabilities and opportunities of single-molecule fluorescence microscopy techniques and potentially help adopt these techniques to further our mechanistic understanding of HR.

Repairing dsDNA breaks involves three phases—pre-synaptic, synaptic, and post-synaptic—corresponding to the trimming of the dsDNA backbone creating single-stranded DNA (ssDNA) overhangs, formation of a homologous exchange intermediate called a Holliday junction (HJ), and, finally, the resolution of the junction back into dsDNA [1,7]. A number of enzymes are responsible for performing one or more roles in these processes for successful execution of DNA break repair. However, due to the involvement of the same enzymes in more than one specific function, there is still a limited understanding of their roles in the HR process.

**Figure 1:**
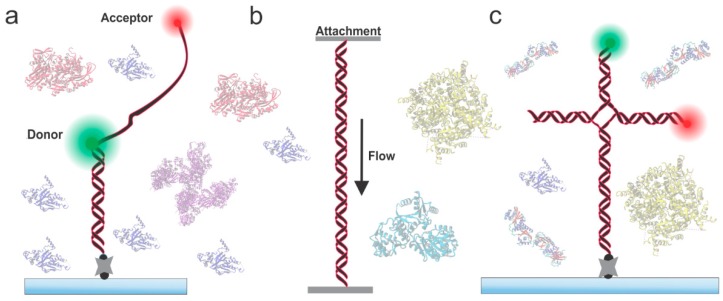
Three key DNA substrates (i.e., single-stranded DNA (ssDNA), double -stranded (dsDNA), and Holliday Junction (HJ)) of the homologous recombination (HR) process. (**a**) Typical fluorescence resonance energy transfer (FRET) based DNA construct designed to study ssDNA-binding proteins. These constructs generally consist of a dsDNA region affixed to the surface of a microscope slide and a flanking ssDNA region for protein binding. The constructs are functionalized with a pair of fluorophores (donor and acceptor) to enable FRET-based imaging and to monitor protein binding. (**b**) Generalized representation of dsDNA curtain, wherein a dsDNA is stretched between two micro-fabricated attachment points. Fluorescently labeled proteins can then be used to study binding. (**c**) Surface-bound HJ analog used in FRET-based imaging. Structures on the background are the crystal structures of some of the HR proteins [12,13,14,15,16,17,18].

The groundwork towards unraveling complex pathways of the HR process have been laid out initially through a number of biochemical studies. The protein–DNA complexes that form during the HR process have been revisited more recently via single-molecule approaches and have significantly improved our understanding of the process [19,20,21,22,23,24,25]. For example, the bulk biochemical work by West’s group [24,26,27] and Ogawa’s group [28,29] provided a better understanding of structure–function relationships of several HR enzymes. However, due to the inherent averaging of these bulk assays and inability to reveal dynamic aspects of molecular interactions, these studies provided only limited information. Monitoring of real-time biomolecular interactions typically requires a more direct approach such as high-resolution microscopy. Recent advancement in fluorescence microscopy approaches presents a powerful tool to enable direct visualization of protein–protein and protein–DNA interactions that are critical to HR. In fact, because these techniques bypass ensemble averaging, they allow real-time visualization of the activity of individual fluorophore-labeled molecules, ultimately providing unprecedented insights into the subcellular world that would otherwise not be possible to access. For example, a fluorescence resonance energy transfer (FRET) based approach provides a signal based on the distance between the donor and acceptor fluorophore (Figure 1A) and, thus, it has been used to study HR proteins acting on DNA [30,31]. Using simple fluorescence-based imaging, both single- and double-stranded DNA curtains have been developed to study filament-forming proteins (Figure 1B). By using a fluorescently labeled HJ structure, the binding of HJ resolvases have been probed (Figure 1C).

Various protein systems that are involved in the repair of dsDNA breaks via the HR pathway have several members, some of which act on both the dsDNA and ssDNA substrates. For instance, RecBCD is a complex of three enzymes which cuts and separates an ssDNA from genomic dsDNA at the chi sequence, leaving in its wake a trail of RecA protein bound to the ssDNA. The creation, protection, and repairing of the ssDNA segments is essential in the dsDNA break repair pathway. Typically, the ssDNA segments are protected by single-stranded DNA binding proteins (SSBs), where the SSBs wrap around the ssDNA portions to protect them from other cellular processes [32] and facilitate their roles in HR. For example, although RecA is mostly known for its ssDNA-binding activity, it plays a key role in synapsis by binding to homologous dsDNA [33]. The RecA homolog, Rad51, serves a similar role in humans as a key player in the formation of synapsis with both ssDNA and dsDNA binding activity. Another key protein is RecQ, which is a helicase with various roles such as unwinding of dsDNA and promoting DNA annealing [34]. Similarly, the replication protein A (RPA) is an indispensable player in HR among other important DNA metabolic pathways in eukaryotes [2]. Further, the repair of single-strand gaps in dsDNA is accomplished by the enzyme complex RecFOR using a homologous mechanism [35,36,37]. On the other hand, the resolution of the HJ cross-strand intermediate formed among the homologous DNA molecules during the late-stage of HR is recognized and resolved by the protein complex RuvABC (composed of three proteins RuvA, RuvB, and RuvC) in prokaryotes [27] and different sets of proteins in eukaryotes. In eukaryotes, the RuvABC protein complex builds upon the HJ in a sequential manner with RuvA locating and binding to the HJ, RuvB binding to the RuvA-HJ complex and initiating branch migration, and RuvC later binding to and resolving the HJ [38]. As we work through this review, some of the proteins will appear in multiple sections, as they have both ssDNA- and dsDNA-binding activities. In this review, we discuss these recent advances in the HR field that have harnessed the unique ability of single-molecule fluorescence microscopy to reveal unprecedented molecular-level details of critical protein–DNA complexes.

## 2. Probing HR Proteins Operating on ssDNA

The ssDNA-binding HR proteins have largely been explored using single-molecule FRET (smFRET) or DNA curtain platforms. The DNA constructs used in smFRET studies generally follow a simple design as shown in Figure 1A, wherein a region of dsDNA is bound to the surface of the microscope slide with a short biotin-modified ssDNA overhang [39,40,41,42]. The construct is generally labeled with a single fluorophore to enable fluorescence imaging or a donor–acceptor pair to enable FRET (Figure 1). The FRET-labeled constructs are particularly suitable for studying filament-forming HR proteins (e.g., Rad51 and RPA) that bind to the exposed single-stranded region and modify its length, shape, and/or stiffness [39,40,41,42]. The FRET techniques, being sensitive to sub-nanometer distance changes between the donor and acceptor fluorophores [30,31], allow any change in the ssDNA conformation or length to be directly translated to FRET. This powerful yet simple technique can provide unique insights into the nature of binding and unbinding of proteins. The DNA curtain techniques, on the other hand, use linearized pieces of DNA anchored on a fixed platform on one or both ends while fluorescently-labeled species of interest, such as HR proteins, assemble upon them [43]. These techniques can utilize ssDNA or dsDNA molecules, and both of them will be discussed in this review. The assembly and activity of these differently labeled proteins of interest can be studied using fluorescent colocalization, a technique where the emissions of two or more species are tracked in space, and the relative localization and motion of these species are used to infer interactions and/or function. Unlike smFRET, where at least two different types of fluorophores (acceptor and donor) are used and the ratio of emissions of the donor and acceptor are monitored to determine the FRET efficiency (and ultimately the donor–acceptor distance), emissions in space are used to determine the locations of molecules in the colocalization experiments. Some of the key advantages of the DNA curtain platform are the ability to carry out highly parallelized localization experiments of DNA binding/processing protein(s) on single extended DNA molecules using proteins tagged with fluorophores and/or DNA-specific fluorescent dyes [43]. Below, we discuss recent single-molecule studies of HR proteins operating on ssDNA.

Single-strand DNA binding proteins (SSBs) and their analogs are essential in protecting ssDNA regions in various biological processes such as preventing the formation of ssDNA secondary structures and assisting in recruiting certain RecA mediators [32]. The displacement of SSBs from ssDNA substrate is one of the first steps in homologous recombination. This is generally accomplished by RecA displacing the protein through formation of its own filaments around the ssDNA [32]. Bell et al. [44], in 2015, studied the assembly of fluorescently labeled SSBs onto ssDNA using a DNA curtain technique where ssDNA molecules that were tethered at one end were extended by a gentle buffer flow and visualized using fluorescence microscopy. This study determined that the ssDNA is wrapped around SSB tetramers in 30–70 nucleotide sections, and that SSB exchanges with the solution protein regularly [44]. A study by Joo et al. [39], in 2006, further clarified the reported interaction between SSB and RecA, where the filament-forming ability of RecA on SSB-saturated DNA was measured using a FRET system. This study showed that RecA displaces SSB at normal filament formation rates as long as this displacement travels outward from a pre-formed RecA nucleation site, demonstrating that no active mechanism to clear SSB off ssDNA is required [39]. The direct binding of human SSB analog SSB1 in complex with RPA to ssDNA was demonstrated using single-molecule FRET in 2013 by Yang et al [41]. This study found that the SSB1 complex binds to ssDNA with a lower affinity in the absence of RPA [41]. Bell et al. [37] further explored the interaction between SSB and RecA, in 2012, using a variant of the DNA curtain method, where surface-tethered SSB-bound ssDNA molecules were extended linearly using a gentle flow and then exposed to fluorescently labeled RecA. The rate and nature of RecA filament formation in SSB-bound conditions was then monitored. This study demonstrated that the RecA nucleation starts at various points along the strand with the binding of a dimer and radiates out from these points of nucleation in either direction with some preference for 5’ to 3’ filament growth. This study also showed that the RecA nucleation is facilitated by RecFOR, and the speed of filament growth is enhanced by RecOR [37].

**Figure 2:**
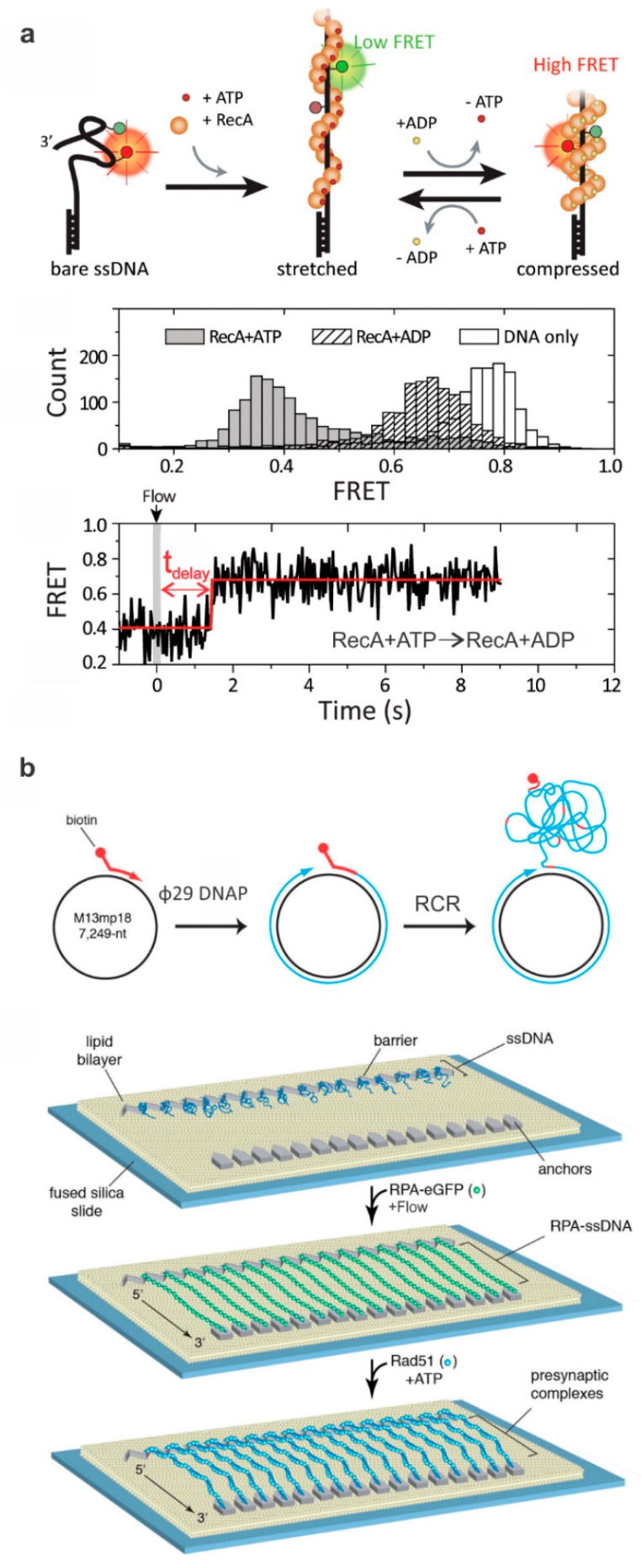
Typical DNA constructs to study ssDNA-binding proteins. (**a**) The formation of Rad51 filament around the ssDNA changes FRET efficiency due to ATP-dependent extension of the ssDNA (middle panel). The bottom panel shows a typical single-molecule FRET-time trace illustrating the delay between the buffer exchange and conformational switch from the stretched to compressed states. This figure is reprinted with permission from Kim et al. [40] and any further inquiries for permissions should go to ACS. (**b**) Depicted is a procedure for making ssDNA curtains and a schematic of the rolling circle replication (RCR) to produce ssDNA molecules for ssDNA curtains using a biotinylated primer, M13mp18 circular DNA, and Φ29 DNA polymerase (Φ29 DNAP). The ssDNA molecules are anchored to a surface coated with a lipid bilayer; flow is then used to extend the DNA which then anchors to downstream barriers non-specifically. Green fluorescent protein (GFP)-labeled RPA then assembles in a filament around the ssDNA, which can be visualized using fluorescence microscopy. In this specific example, a mixture of Rad51 and ATP was incubated with the curtain and Rad51-mediated displacement of GFP-RPA was monitored. Adapted with permission from Elsevier [43].

The properties and functions of RecA filaments have been the subject of major interest for single-molecule fluorescence studies in various stages of the HR process [45]. In 2012, Ragunathan et al. [42] studied RecA’s role in homology search using a FRET system with an acceptor fluorophore integrated into the ssDNA portion of the DNA construct and a donor fluorophore integrated on the dsDNA. They found that the dsDNA “slid” across the RecA filament at a speed that allowed more DNA to be sampled in homology search than previously suspected [42]. Kim et al. [46] studied the effect of sequence on the formation of the RecA filament using a FRET construct with fluorophores attached among sequences of interest. This group found that the chi sequence (5’-GCTGGTGG) does not itself promote nucleation, but that decreased monomer exchange due to the RecA’s affinity for TGG repeats increases the amount of time that RecA pauses at this sequence, promoting nucleation [46]. Kim et al. [40] showed that the RecA filament switches between a relaxed and stretched conformation in the presence of adenosine di-phosphate (ADP) and adenosine tri-phosphate (ATP), respectively (Figure 2A). This switching between relaxed and stretched conformations allows the filament to remain assembled during various stages of HR. This is due to the model of the filament stretching and relaxing cycle, in which cooperative structural changes within adjacent RecA monomers allow the filament to stay in its current state in the midst of ATP exchange [40].

The Rad51 nucleation was studied using an ssDNA construct with a FRET pair on the single-stranded region by Lu et al. [47]. This study found that accessory factors, Swi5-Sfr1, stabilize the Rad51 monomers allowing for an enhanced filament formation [47]. In 2012, Gibb et al. [43,48] established a technique for visualizing DNA-binding proteins on an ssDNA curtain. In this method, ssDNA molecules created through a process called rolling circle replication (RCR) are attached to biotinylated lipids that form a part of a lipid bilayer coating on a slide surface. The ssDNA molecules are lined up on a nanofabricated ridge and then extended using a buffer flow (Figure 2B) [43,48]. Gibb et al. [49] used this platform in 2014 to anchor dsDNA curtains to study the presynaptic interplay of yeast RPA, Rad51, and Rad52. Using fluorescently labeled proteins, they determined that Rad52 binds to RPA and prevents RPA unbinding. This result implicated that a RPA–Rad52–Rad51 intermediate has a significant role in late-stage HR [49]. In 2017, Ma et al. [50] used this approach to study the assembly and disassembly of Rad51 filaments. In this study, the rate of displacement of a fluorescently labeled RPA was used to determine that the presence of RPA in solution inhibits Rad51 filament formation but not extension. This result suggests that RPA has a role in Rad51 regulation and potentially modulates the properties of already assembled Rad51 filaments [50]. Myler et al. [51] used an ssDNA curtain technique and fluorescently labeled proteins to determine the effect of RPA on ExoI binding. Using fluorescent colocalization microscopy, they showed that ExoI binding was interrupted by the binding of RPA and that SSB1 enhanced the binding of ExoI. This result indicates that SSB1 and RPA have a significant role in regulating the resection of DNA by Exo1 in DNA damage repair in humans [51].

In addition, the ssDNA platform has also been used to study other HR proteins. For example, in 2017, De Tullio et al. [52] used ssDNA curtains to study the assembly of Srs2 onto Rad51-bound ssDNA. This study found that Srs2 moves along the DNA unidirectionally, displacing Rad51 in an ATP-dependent manner. When Rad52 was introduced and allowed to bind to the Rad51 filament, it had no effect on the ability of Srs2 to displace the Rad51 from ssDNA [52]. Later, the same group found that Srs2 displaces Rad51 in a unidirectional manner, and that Srs2 begins this displacement from points where RPA has already bound, clearing the way for more RPA molecules which then recruit more Srs2 molecules [53]. The binding of Rad52 to both ssDNA and dsDNA was explored using an optical tweezers system and fluorescently labeled Rad52 in 2017 by Brouwer et al [54]. While Rad52 was found to have a strong and static binding with ssDNA, it binds to dsDNA less tightly in a transient manner. Using force spectroscopy, they were also able to determine that Rad52 helps stabilize dsDNA to prevent melting, which could be advantageous during certain steps of recombination [54]. In 2019, Crickard et al. [55] explored the relationship between highly similar proteins RPA and Dmc1 both of which are present in cells during meiotic HR and have very similar binding behaviors. Using fluorescently labeled proteins, they determined that, while both proteins form filaments under similar conditions, Dmc1 binds specifically to the Hop2–Mnd1 complex that has been implicated in recruiting dsDNA to the synaptic complex during meiotic HR [55].

## 3. Probing HR Proteins Operating on dsDNA

Investigation of dsDNA-binding HR proteins has been an area of considerable work in the fluorescence microscopy field. These proteins are critical to the presynaptic and synaptic stages of HR and often work in concert. Therefore, bulk methods have difficulty analyzing these complex interactions. Studies investigating and visualizing the binding of these HR proteins are by large performed on some linearized piece of dsDNA molecules hold in place under a fluorescence microscope. The mechanism by which the dsDNA molecules are held in place can be a “DNA curtain” approach, where the dsDNA molecules are bound to a functionalized slide surface on one end with continuous buffer flow to hold the DNA linear or are bound on both ends. Another method for holding a dsDNA molecule linear is binding the DNA to a functionalized polystyrene bead which is then held in a moveable optical trap, using a flow to keep the DNA extended. Exposure of DNA to different buffers is facilitated by physically moving the bead into various areas of a flow cell with buffers separated by laminar flow.

**Figure 3:**
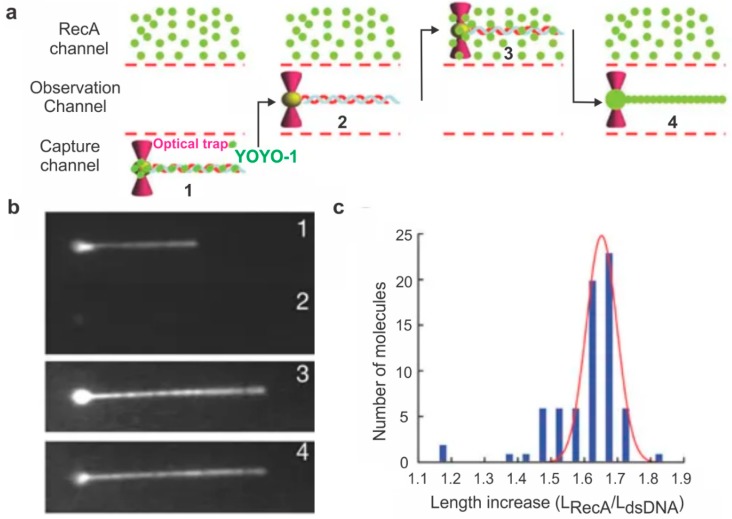
Optical tweezers-based monitoring of RecA assembly on dsDNA. (**a**) Experimental setup used to study the effect of RecA on dsDNA length. Biotin-functionalized lambda DNA stained with a DNA-binding dye called YOYO-1 was attached to a bead held in a moveable optical trap. This DNA was held linear with a constant laminar flow, and the bead was moved to various channels (shown by arrows) either to expose the DNA to protein or to visualize it. (**b**) The DNA was first located, and its length was determined. The bead was then moved to the observation channel where YOYO-1 dissociates from the DNA and then to a channel containing fluorescently labeled RecA. The length of the DNA was again recorded after RecA binding. Images of the positions of the DNA bead complex as described in (**a**). (**c**) Histogram showing the distribution of the length change of DNA with (*L*_RecA_) and without (*L*_dsDNA_) RecA. This figure is reprinted with permission from Springer Nature [45], copyright Springer Nature 2006.

The RecA filament formation was studied in 2006 by Galletto et al. [45] using dsDNA held in a movable optical trap. This dsDNA was exposed to fluorescently labeled RecA in various concentrations, and the binding was tracked using a fluorescence microscope. This strategy allowed them to determine that the length of dsDNA increased by ~70% upon RecA filament formation (Figure 3). They also showed that the RecA nucleation was dependent on the presence of a nucleoside triphosphate but the filament growth after nucleation could occur bidirectionally around the nucleation site with or without the cofactor [45]. In 2012, Forget et al. [56] used an optical trap system to study the physical nature of RecA’s role in homology search, a key step in HR. In this study, they used fluorescently labeled RecA and, by monitoring the end-to-end distance of the dsDNA molecule, they demonstrated that the rate of homology search varies with the degree of linearization of the DNA molecule. They attributed this observation to a 3D homology search mechanism where the RecA protein samples 3D space around it rather than linearly down a single DNA molecule, a mechanism they called intersegmental contact sampling. According to this model, the RecA filament has a polyvalent surface which can sample several non-contiguous parts of adjacent dsDNA; this in combination with the globular nature of dsDNA under relaxed conditions allows the RecA filament to sample numerous nearby neighboring sequences during its search for homology [56].

In 2001, Bianco et al. [57] published a study visualizing the DNA unwinding activity of the RecBCD enzyme complex. In this study, they bound an end-functionalized dsDNA molecule to a surface-functionalized polystyrene bead and held the bead in an optical trap. The DNA was then exposed to a DNA-binding fluorescent dye called YOYO-1 to visualize the DNA. This fluorescent DNA was exposed to the RecBCD complex which processed through the DNA releasing the florescent dye thus allowing the direct measurement of the rate of RecBCD procession along the DNA. This study demonstrated that the unwinding of RecBCD proceeds continually at ~0.3 µm s^−1^ until this protein complex dissociates [57]. This work was also complemented by the crystal structure of RecBCD obtained by Singleton et al. [58]. In 2012, Yang et al. [59] studied RecBCD’s affinity for the chi sequence more closely using the same technique as Bianco et al. [57]. In this study, they showed that the specificity of RecBCD towards the chi sequence was either compromised or completely abolished when a suspected chi-recognition domain was mutated [59]. In 2013, Liu et al. [60] used optical tweezers to study the variation in rates of RecBCD’s unwinding kinetics. In this study, processing of DNA by RecBCD was interrupted by moving the optical trap to a buffer channel containing no ATP and then moved back to the ATP-containing channel to reinitiate the RecBCD activity. Using this approach, they observed that the DNA unwinding rate of individual enzyme molecules varies. They attributed this observation to the ATP-dependent stabilization of one of many equilibrium sub-states, each of which has a slightly different conformation and, thus, exhibits a different progression rate [60].

The essential HR protein, Rad51’s, filament-forming behavior was studied in 2006 by Prasad et al. [61] using a DNA curtain platform wherein the length of YOYO-1 labeled dsDNA was monitored before and after binding of Rad51. As Rad51 bound to the dsDNA, the overall length of the DNA molecules increased due to the extended conformation of DNA upon formation of the Rad51 filament. Further, after systematic mutation of Rad51 in various domains, it was found that the protein’s L1 domain was essential for filament formation [61]. In the same year, the Greene [62] lab developed a new platform for studying protein–DNA interactions, called DNA curtain, in which several dsDNA molecules are stretched and attached on both ends creating a parallel array of DNA molecules. The parallel array of DNA molecules can be visualized via total internal reflection fluorescence (TIRF)-based single-molecule fluorescence microscopy by directly staining the DNA molecules with fluorescent dyes. On the application side, protein–DNA interactions can be studied on the DNA curtain platform using fluorescently tagged proteins. For example, Yeykal et al. [62] used this method to show that a GFP-tagged Rad51 can diffuse along the dsDNA molecules in a one-dimensional random walk using only Brownian forces.

**Figure 4:**
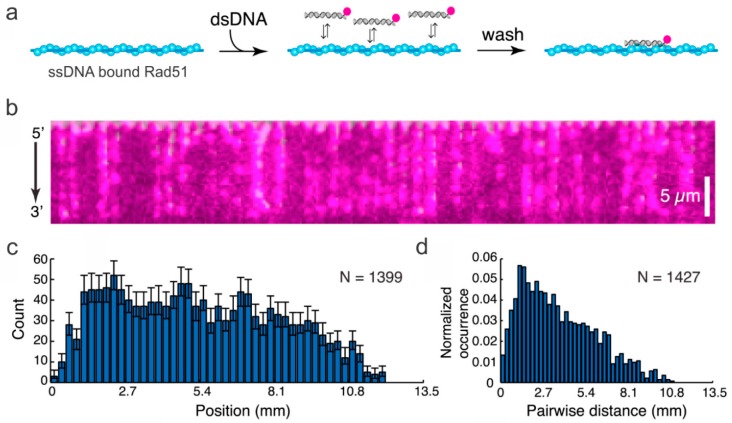
The ssDNA curtain used to study the progression of dsDNA fragments along a Rad51 filament. (**a**) The strategy for visualizing dsDNA binding to the Rad51-modified ssDNA. The dsDNA was fluorescently labeled. (**b**) The DNA curtain displaying the binding of dsDNA on Rad51-modified ssDNA. (**c**) The binding site distribution on the DNA measured from the 5′-end of the DNA and (**d**) the distance between fluorophores on the Rad51 filament. This figure was reprinted with permission from Elsevier [63], copyright 2015.

The double-bound ssDNA curtain (Figure 4) provided an exceptional platform for investigating the homology search function of Rad51. In 2015, Qi et al. [63] used this method to study the progression of fluorescently tagged homologous dsDNA along a Rad51 filament built up around the curtain. They were able to determine that Rad51 uses a length-based mechanism that looks in segments of 8 nucleotides to speed up the search process [63]. In another application, Rad et al. [64] used a dsDNA curtain system to visualize the helicase unwinding activity of RecQ. In this study, they showed that, as RecQ unwinds the dsDNA, the fluorescently labeled SSB binds. This system visualized not only the rate and number of ssDNA regions created upon RecQ exposure but also that the ssDNA forks were formed upon exposure. More interestingly, they were able to determine that RecQ dimers are required to initiate this unwinding process and that unwinding proceeds at a rate of 40–60 bp s^−1^ [64].

## 4. Probing HR Proteins Operating on Holliday Junctions

Studying proteins that bind to the HJ using single-molecule microscopy presents interesting challenges and opportunities due to the inherent conformational changes that occur in the HJ [27,65,66]. The HJ is a naturally dynamic structure that switches between two stacked conformers many times per second through an open cruciform intermediate [65]. In recent years, the dynamic nature of the junction has been exploited to probe the interaction between the junction and HR proteins. This is generally done by attaching a pair of fluorophores on the ends of adjacent arms of the HJ to enable FRET. Due to the dynamic nature of the HJ, it results in a distinct FRET efficiency for each conformer.

Single-molecule studies of the HJ have determined the structural dynamics of the HJ, particularly the shape that the junction adopts and the circumstances (primarily ionic environment and nucleotide composition at the core of the junction) under which it would switch from one stacked conformer to another. In 2003, McKiney et al. [65] used an HJ labeled with a FRET pair on adjacent arms and a Ca^2+^-rich buffer to determine the energy landscape which governs the switching of the HJ between its stacked conformers. They concluded that the conformational switch must occur during many intermediate stages of HR [65]. The stepwise random progression of branch migration (the migration of the four-way junction along the DNA axis) was experimentally visualized by Karymov et al. [67] in 2005. This study showed that the junction switches between its stacked and cruciform conformations; the cruciform being more favorable to branch migration, as it allows the HJ to migrate several base pairs at once. They also found that, as the concentration of Mg^2+^ increased, the HJ switched to its stacked conformation more often, stalling the branch migration [67]. The relative abundance of the HJ cruciform intermediate in solution was determined by Joo et al. [68]. They also proposed that this cruciform conformation could potentially be stabilized through protein binding [68]. The conformational switching of the HJ was further studied by Hohng et al. [69] using a surface-bound and bead-tethered HJ labeled with a FRET pair. This system allowed the researchers to apply force to the HJ and monitor its conformation via FRET signal. In this manner, they were able to map two-dimensional energy landscapes of conformational switching of the HJ [69]. The binding interaction of the MutSγ analog Msh4-Msh5 to HJs and other types of DNA structures was probed in depth by Lahiri et al. [70,71] in 2018, discovering that the MutSγ stabilizes the HJ and prevents branch migration until resolution by MutLγ [70,71].

**Figure 5:**
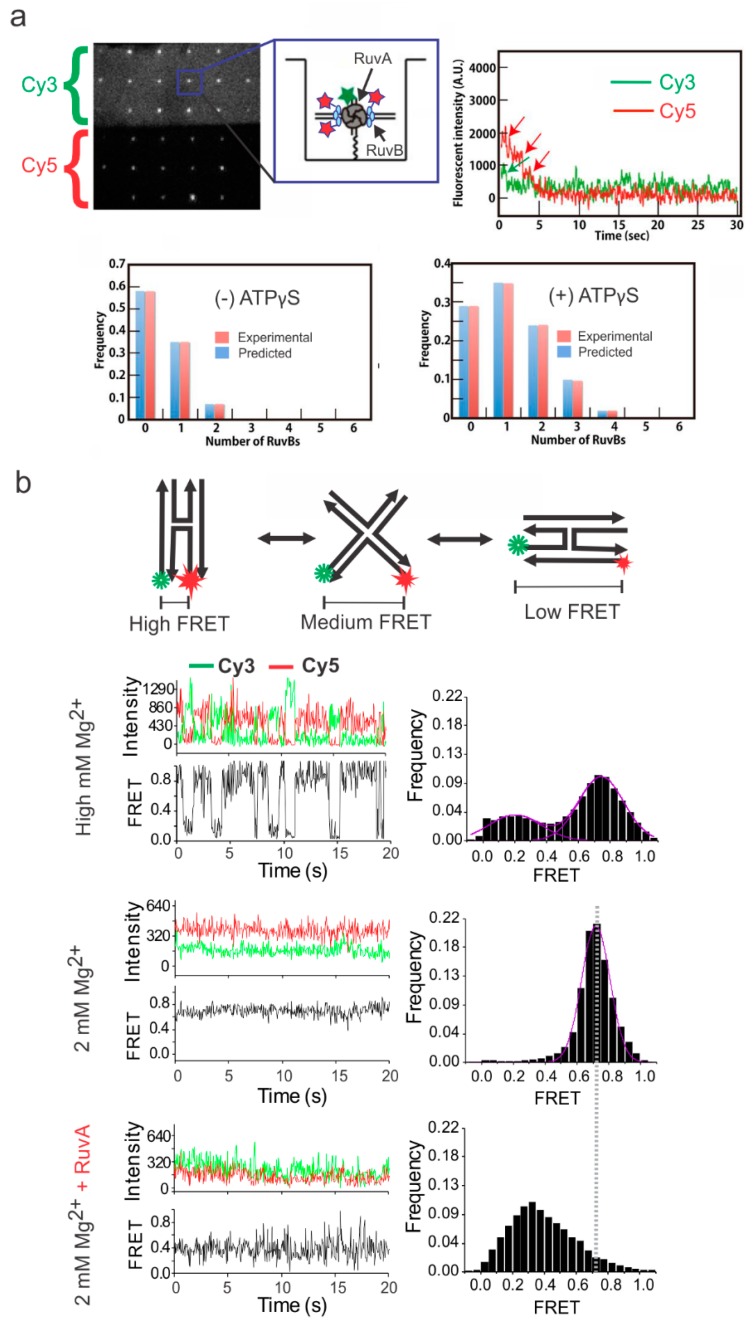
Probing the binding interaction between HR proteins and HJ. (**a**) Single-molecule waveguide experiment on a RuvAB–HJ complex. Cy3 and Cy5 channels represent the fluorescence emission from these respective fluorophores. Inset depicts a RuvAB–HJ complex, where RuvA is labeled with a Cy3 fluorophore (green), and RuvB is labeled with a Cy5 fluorophore (red). The top right panel depicts the photobleaching events for counting the number of RuvA and RuvB molecules in the complex. The bar graphs demonstrate the number of RuvBs determined based on the photobleaching events in the absence and presence of ATPγS. This figure was adapted with permission from Iwasa et al. [72]. (**b**) Probing the binding of RuvA to the junction using single-molecule TIRF microscopy. Top panel: Schematic of HJ dynamics between two isomers via an open-state conformation. At high Mg^2+^ concentration without RuvA, the switching between the two isomers was clearly captured (typically >50 mM Mg^2+^ is known to slow down the switching). The occupancy of the low (~0.2 FRET) and high (~0.8 FRET) FRET efficiency states were at ~70:30 distribution. Decreasing the Mg^2+^ concentration to 2 mM, the rapid switching between the two stacked conformations resulted in weighted average of the FRET efficiency of ~0.7. Interestingly, when the HJ was exposed to RuvA, the FRET efficiency shifted to ~0.38, demonstrating that RuvA clamps the HJ in its open conformation. This figure was adapted with permission from the American Chemical Society [66], copyright (2018) American Chemical Society.

Overall, the direct visualization of HJ-binding proteins is an ongoing research effort with only a few HR proteins having been studied. In 2015, Iwasa et al. [72] investigated the assembly of RuvB onto a RuvA-bound HJ using fluorescently tagged RuvB and HJ (Figure 5A). In this study, through the counting of fluorescence photobleaching events, they were able to determine that six RuvB molecules assemble around the DNA arm of the HJ. They also determined that the RuvB’s assembly around the RuvA-bound HJ was an ATP-dependent process [72]. The geometry of the RuvA-bound HJ was studied using a FRET-labeled HJ construct in 2018 by us [66]. In this study, we demonstrated that, upon binding to the junction, RuvA ceases the dynamics of the HJ. The observed FRET efficiency of the RuvA–HJ complex indicated that the RuvA traps the HJ in its open cruciform conformation. Additionally, experiments at various concentrations of Mg^2+^ and Na^+^ showed a strong relationship between the ion concentration and RuvA binding activity (Figure 5B), with a compromised binding at higher concentration of Mg^2+^, suggesting that the RuvA–HJ interaction is primarily electrostatic in nature [66].

**Figure 6:**
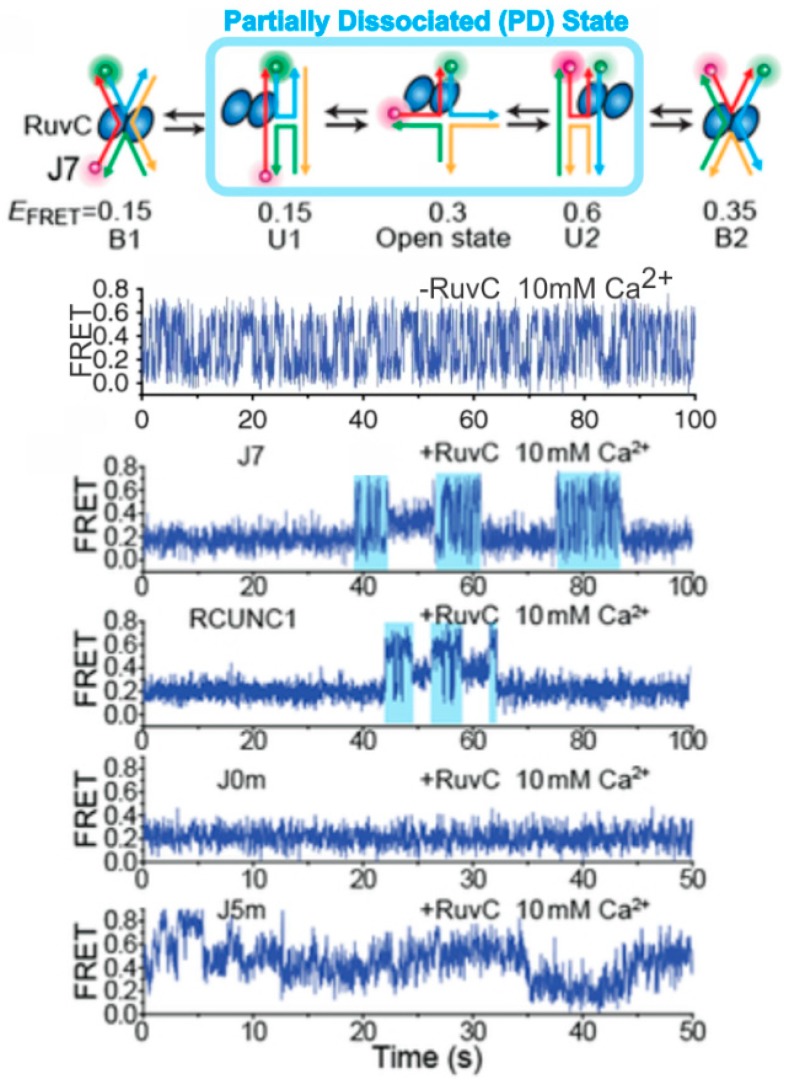
Single-molecule probing of RuvC–HJ interaction. The HJ resolvase RuvC binds to the HJ in such a manner that partial dissociation can occur, where one of the two RuvC dimers unbinds allowing the HJ to switch between its stacked conformations before being bound again by both dimers in one of the two stacked conformations. The single-molecule FRET traces correspond with, in descending order: no enzyme with a rapidly switching HJ; HJ J7 with RuvC, showing partially dissociated condition in cyan; HJ RCUNC1 with RuvC, showing the same partial dissociated (PD) state; J0m with RuvC showing no PD; and HJ J5m with RuvC. J7, RCUNC1, J0m, and J5m represent HJ with different core nucleotide sequences. The figure was adapted with permission from Nature Publishing Group [73], copyright Springer Nature 2019.

In 2019, Zhou et al. [73] used FRET-labeled HJs to study the binding of late-stage HJ resolvases. In this study, the group used a high ionic strength buffer composed of calcium chloride to slow down the conformational switching of the HJ without interfering with the RuvC binding. Using this system, they studied the binding of T7 endonuclease 1, RuvC, GEN1, and hMus81-Eme1 to the HJ and found that they all shared the same dynamic HJ activity while bound. They concluded that these enzymes, all dimers, use multivalent binding to allow the HJ to freely engage in conformational switching (Figure 6), revealing several interesting mechanistic roles of these enzymes which had thus far not been explored [73].

## 5. Discussion and Future Perspectives

In this review, we discussed recent work in the area of homologous recombination focusing on single-molecule fluorescence microscopy which has propelled our understanding of how HR proteins repair DNA breaks. More specifically, we described recent efforts to visualize how HR proteins operate on various DNA substrates—ssDNA, dsDNA, and Holliday junctions. We provided an overview of the methods used in these studies including FRET-based single-stranded DNA constructs, both single- and double-stranded DNA curtains, optical tweezers, and FRET-based analysis of Holliday junction analogs. These studies provided insights into the mechanism of protein binding, determined the kinetics of filament formation by various ssDNA-binding proteins, and revealed regulatory effects of protein–protein interactions on filament formation. More recent studies on HJ-binding proteins have revealed binding modes and effects of ionic environments on the binding interaction. These studies, along with bulk biochemical studies, have also started to decipher how these HR proteins contribute to the formation of branch migration complex and HJ-resolving machinery [67,74,75,76]. It is clear that single-molecule fluorescence microscopy has shed light onto the research that has dealt with the inner workings of homologous recombination machinery. While these studies have significantly improved our understanding of the HR process, due to the involvement of the same proteins in multiple functions and their complex interaction with other proteins for coordinated action, there is still much work to do for a complete understanding of the kinetics and mechanism of the HJ resolution process.

In summary, using fluorescence microscopy, significant progress has been made in understanding the roles individual HR proteins play in the overall HR process. Some inroads have been made in determining the exact roles the HR proteins play on various stages of HR. However, an understanding of the synergistic effect that multiple HR proteins have on the HR process has not yet been fully achieved. Again, more work needs to be done to characterize the way different HR proteins work together to execute the entire HR process. Particularly, the steps and proteins involved in the transition from the synaptic to post-synaptic phases in human HR need more attention. To address these knowledge gaps, several labs around the world are using fluorescence microscopy and other forms of single-molecule techniques to dissect the HR process in detail. Combining the bulk biochemical work along with the molecular-level mechanistic insights obtained from single molecule work, we envision that we will have a complete picture of HR in the near future. Once this complete picture is established, it will significantly enhance our understanding of the HR process in the context of DNA break repair, and, in the long-term, it will become possible to intelligently target HR proteins for therapies.
